# Impact of Previous Pulmonary Tuberculosis on Chronic Obstructive Pulmonary Disease: Baseline Results from a Prospective Cohort Study

**DOI:** 10.2174/1386207325666220406111435

**Published:** 2023-01-06

**Authors:** Yide Wang, Zheng Li, Fengsen Li

**Affiliations:** 1 Department of Integrated Pulmonology, The Traditional Chinese Medicine Hospital Affiliated to Xinjiang Medical University, Urumqi, 830000, P.R. China;; 2 National Clinical Research Base of Traditional Chinese Medicine in Xinjiang, Urumqi, 830000, P.R. China

**Keywords:** Chronic obstructive pulmonary disease, tuberculosis, dose-response relationship, China, cohort study, COPD

## Abstract

**Objective:**

Pulmonary tuberculosis (PTB) is a significant risk factor for COPD, and Xinjiang, China, has a high incidence of pulmonary tuberculosis. The effects of tuberculosis history on airflow restriction, clinical symptoms, and acute episodes in COPD patients have not been reported in the local population. Besides, the exact relationship between lung function changes in people with a history of tuberculosis and COPD risk is not clear.

**Methods:**

This study is based on the Xinjiang baseline survey data included in the Natural Population Cohort Study in Northwest China from June to December, 2018. Subjects' questionnaires, physical examination, and lung function tests were performed through a face-to-face field survey to analyze the impact of previous pulmonary tuberculosis on local COPD. Furthermore, we clarified the specific relationship between pulmonary function decline and the probability of developing COPD in people with a history of tuberculosis.

**Results:**

A total of 3249 subjects were eventually enrolled in this study, including 87 with a history of tuberculosis and 3162 non-TB. The prevalence of COPD in the prior TB group was significantly higher than that in the control group (*p*-value = 0.005). First, previous pulmonary tuberculosis is an essential contributor to airflow limitation in the general population and patients with COPD. In all subjects included, pulmonary function, FEV1% predicted (*p*-value < 0.001), and FEV1/FVC (%) (*p*-value < 0.001) were significantly lower in the prior TB group than in the control group. Compared to non-TB group, FEV1% prediction (*p*-value = 0.019) and FEV1/FVC (%) (*p*-value = 0.016) were found to be significantly reduced, and airflow restriction (*p*-value = 0.004) was more severe in prior TB group among COPD patients. Second, COPD patients in the prior TB group had more severe clinical symptoms. Compared with no history of tuberculosis, mMRC (*p*-value = 0.001) and CAT (*p*-value = 0.002) scores were higher in the group with a history of tuberculosis among COPD patients. Third, compared with the non-TB group, the number of acute exacerbations per year (*p*-values=0.008), the duration of each acute exacerbation (*p*-values=0.004), and hospitalization/patient/year (*p*-values<0.001) were higher in the group with a history of tuberculosis among COPD patients. Finally, a dose-response relationship between FEV1/FVC (%) and the probability of developing COPD in people with previous pulmonary TB was observed; when FEV1/FVC (%) was < 80.8, the risk of COPD increased by 13.5% per unit decrease in lung function [0.865(0.805, 0.930)].

**Conclusion:**

COPD patients with previous pulmonary tuberculosis have more severe airflow limitations and clinical symptoms and are at higher risk for acute exacerbations. Furthermore, lung function changes in people with a history of tuberculosis were associated with a dose-response relationship with the probability of developing COPD.

## 1 INTRODUCTION

Chronic obstructive pulmonary disease (COPD) is a common, preventable, and treatable disease characterized by airflow obstruction. The prevalence of post-bronchodilator COPD is 12.16% (10.91 13.40%) [1]. COPD has become a global public health challenge as it is an important cause of the increase in worldwide mortality and a considerable consumption and expenditure of health resources [2-6]. A growing number of studies have shown that pulmonary tuberculosis (PTB) is a significant risk factor for COPD. In a multicenter study conducted in Latin America, the TB group (FEV1/FVC < 0.7) had a significantly higher rate of flow obstruction than the non-TB group (30.7% *vs*. 13.9%) [7]. In a cohort study conducted by Lam, KB et al., the history of tuberculosis was an independent risk factor for flow obstruction in the population (odds ratio = 1.37; 95% CI, 1.13-1.67), and it was suggested that the high incidence of COPD in the survey sites might be related to the high prevalence of tuberculosis [8]. The interleaved and interconnection between TB and COPD in epidemiology requires us to more actively understand the interaction and association between TB and COPD in morbidity and disease progression.

About one-third of the world's population is now infected with PTB, causing heavy social and economic impacts [9]. China is a country with a high tuberculosis burden, accounting for about 9 percent of the world's total tuberculosis cases [9, 10]. Xinjiang Uygur Autonomous Region is located in China's northwest and is one of the regions with a high burden of tuberculosis in China. Unfortunately, there is a lack of research on tuberculosis's effect on COPD in Xinjiang, China. Besides, as an essential diagnostic and evaluation index of COPD, lung function is also a significant predictor of COPD incidence [11, 12]. As an independent risk factor for COPD [13], lung function changes in the population with prior TB and the risk of COPD have not been reported.

Therefore, this study will analyze the impact of tuberculosis history on patients with COPD in Xinjiang, China, based on baseline data from a prospective cohort study. Besides, we will further analyze the specific relationship between pulmonary function changes and the probability of developing COPD among patients with TB history.

## 2 METHODS

### 2.1 Study Subjects

A study on multi-ethnic natural population cohort construction and health follow-up in Xinjiang (2017YFC 0907203) is a sub-project of the “Natural Population Cohort Study in Northwest China”. All the populations included in the study were from this program. Field research was conducted in rural areas of southern Xinjiang, China, from June to December, 2018. Inclusion criteria include: (1) Unrestricted gender, age over 35 years old; (2) No apparent communication or cognitive impairment; (3) Local residents; (4) Complete a valid lung function test; (5) Voluntarily signed informed consent. This study was in accordance with the Declaration of Helsinki. The ethics committee of Xinjiang Uygur Autonomous Region Chinese Medicine Hospital approved this study (IRB ID: 2018XE0108).

### 2.2 Definition of Airflow Limitation

The lung function test was performed using Masterscreen Pneumo (version2.13, Germany). The detection personnel included trained respiratory specialists. The instrument was debugged and calibrated every day before use and then operated. The subjects rested in a quiet state for 15 minutes before the operation; lung ventilation function was measured at least three times for each person, and the best value was retained and recorded. Both COPD diagnostic criteria and GOLD classification criteria are based on the 2017 GOLD Global Initiative on Chronic Obstructive Pulmonary Disease. According to the research conducted by Satia *et al*. [14-16], indicators, such as FEV 1predicted % of lung function, FEV1/FVC (%), and classification of severity of airflow limitation were used in this study to evaluate the severity of airflow limitation of subjects.

### 2.3 Definition of Symptom Severity

In this study, MMRC and CAT scores were used to evaluate the severity of subjects' symptoms [17]. Due to the significant association between mMRC and COPD patients' health status and risk of death, mMRC has become a commonly used questionnaire to evaluate the severity of dyspnea symptoms. However, dyspnea is only one aspect of the symptoms of COPD. Although comprehensive symptom assessment scales, such as SGRQ and CRQ, are more objective, they have the disadvantage of being too complicated. CAT scale has gradually become the quantitative standard for the evaluation of COPD symptoms.

### 2.4 Definition of Acute Exacerbation

By reference to Ko FW research [18], we used the number of acute exacerbations per year, the duration of each acute exacerbation, and hospitalization/patient/year as indicators to evaluate subjects' acute aggravating circumstances.

### 2.5 Definition of Previous Pulmonary Tuberculosis

The subjects were interviewed face to face by trained and qualified investigators. Information about respiratory symptoms, health status, *etc*., was collected. Referring to the studies conducted by Amaral *et al.* [19, 20], previous pulmonary tuberculosis was considered a positive answer to the question: Have you ever been told by a respiratory specialist that you have had tuberculosis? Participants who were on treatment for tuberculosis at the time of the study were excluded from participation.

### 2.6 Statistical Analysis

Quantitative data, such as BMI, waist circumference, and lung function FEV1%, were described by means ± standard deviation. Qualitative data, such as smoking and having COPD, are described by numbers (percentages). The unpaired Student's t-test and the Pearson’s Chi-square test were used to comparing the differences between the two groups. We used the smoothing fitting curve and two-stage regression model to analyze the correlation between the probability of developing COPD and lung function FEV1/FVC ratio (%) among people with pulmonary TB by adjusting for age, economic income, education level, smoke exposure, and other vital covariates, including previous chronic bronchitis, diabetes, insomnia, *etc*.

Data analyses were carried out using Stata Version 14.0 (Stata Corp., College Station, Texas) and Empower (R) (www.empowerstats.com, X & Y Solutions, Inc. Boston, MA). The test level was set at 0.05.

## 3 RESULTS

### 3.1 Study Participants and Characteristics

The basic characteristics of the subjects were described by whether they had previous pulmonary tuberculosis or not (Table **1**). A total of 3249 subjects were included in this study, including 1585 males and 1664 females, with an average age of 52.8±9.4 years. Among them, 87 cases had a history of tuberculosis, and 3162 were in the non-TB group. The incidence of previous pulmonary tuberculosis was 2.7%. There were 394 patients with COPD and 2855 patients without COPD. The prevalence of COPD was 12.1%. The prevalence of COPD in the prior TB group was significantly higher than that in the non-TB group (21.8% *vs*. 11.9%). The history of tuberculosis has significant differences in terms of age, waist circumference, education level, gross annual income, chronic bronchitis, depression, and other factors.

### 3.2 The Associations between Prior TB and Airflow Obstruction

**Fig. (1):**
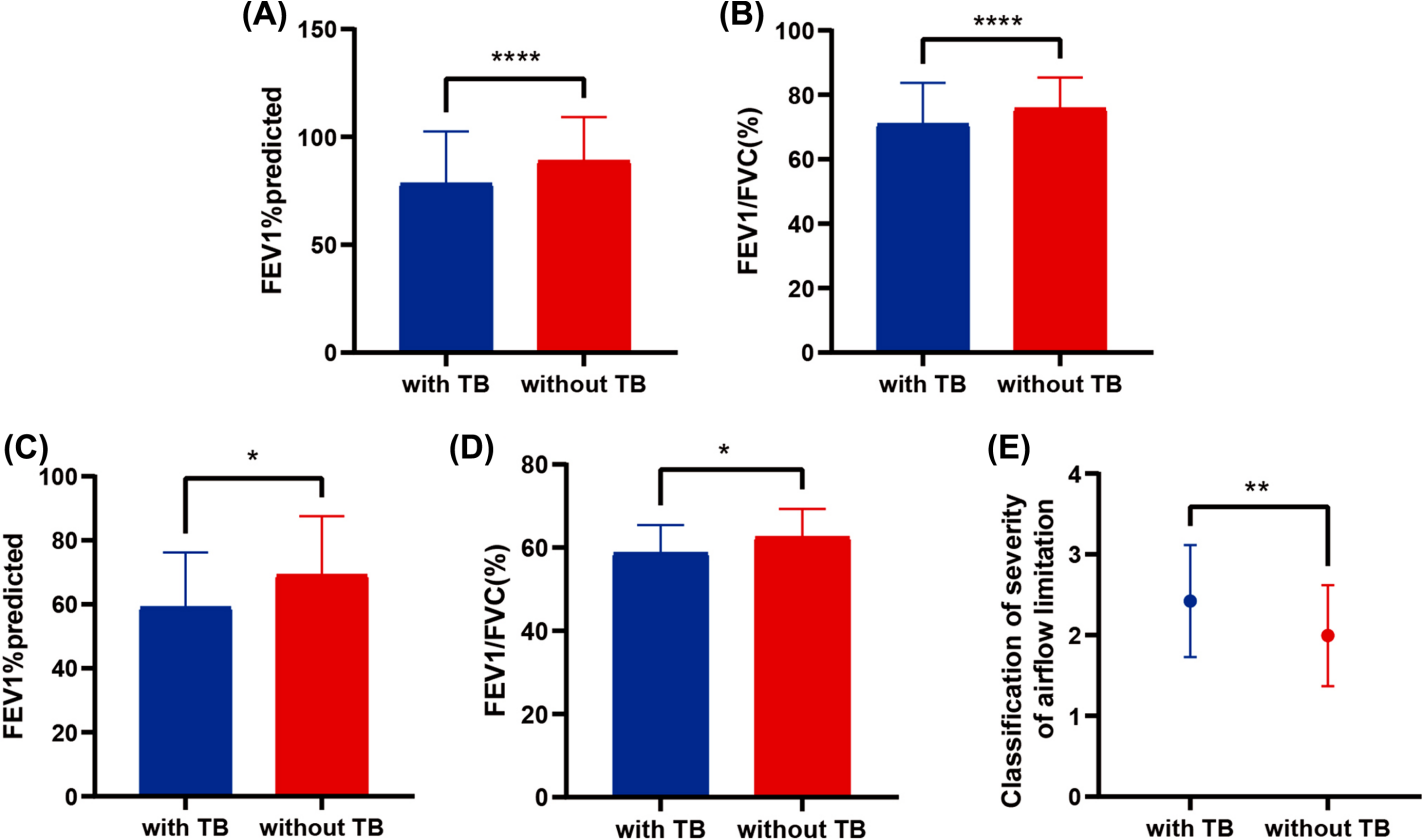
The association between a history of tuberculosis and airflow obstruction. **Notes:** In all subjects included, pulmonary function, including FEV1% predicted (**A**) and FEV1/FVC (%) (**B**), was significantly lower in the group with a history of tuberculosis than in the control group. Compared with the group with no history of tuberculosis, pulmonary function, including FEV1% predicted (**C**) and FEV1/FVC ratio (%) (**D**), was significantly reduced, and airflow restriction (**E**) was more severe in the history of tuberculosis group among COPD patients. **Abbreviations:** TB, pulmonary tuberculosis; FEV1, forced expiratory volume in 1 s; FVC, forced vital capacity.

The association between a history of tuberculosis and airflow obstruction has been observed. In the general population, the index of lung function, including FEV1% and FEV1/FVC, (%), in the prior TB group was lower than those in the non-TB group (Figs. **1A** and **B**), and the difference was found to be statistically significant (all *p*-values<0.001). Compared to the control group of COPD patients, the indicators of lung function, FVC1% predicted and FEV1/FVC (%), in the prior TB group were decreased (Figs. **1C** and **D**), and the differences were statistically significant (*p*-value = 0.019;0.016). Moreover, the classification of severity of airflow limitation in the prior TB group was significantly higher than that in the non-TB group among COPD patients. (Fig. **1E**), and the difference was statistically significant (*p*-value = 0.004).

### 3.3 The Associations between Prior TB and Symptom Severity among COPD Patients

**Fig. (2):**
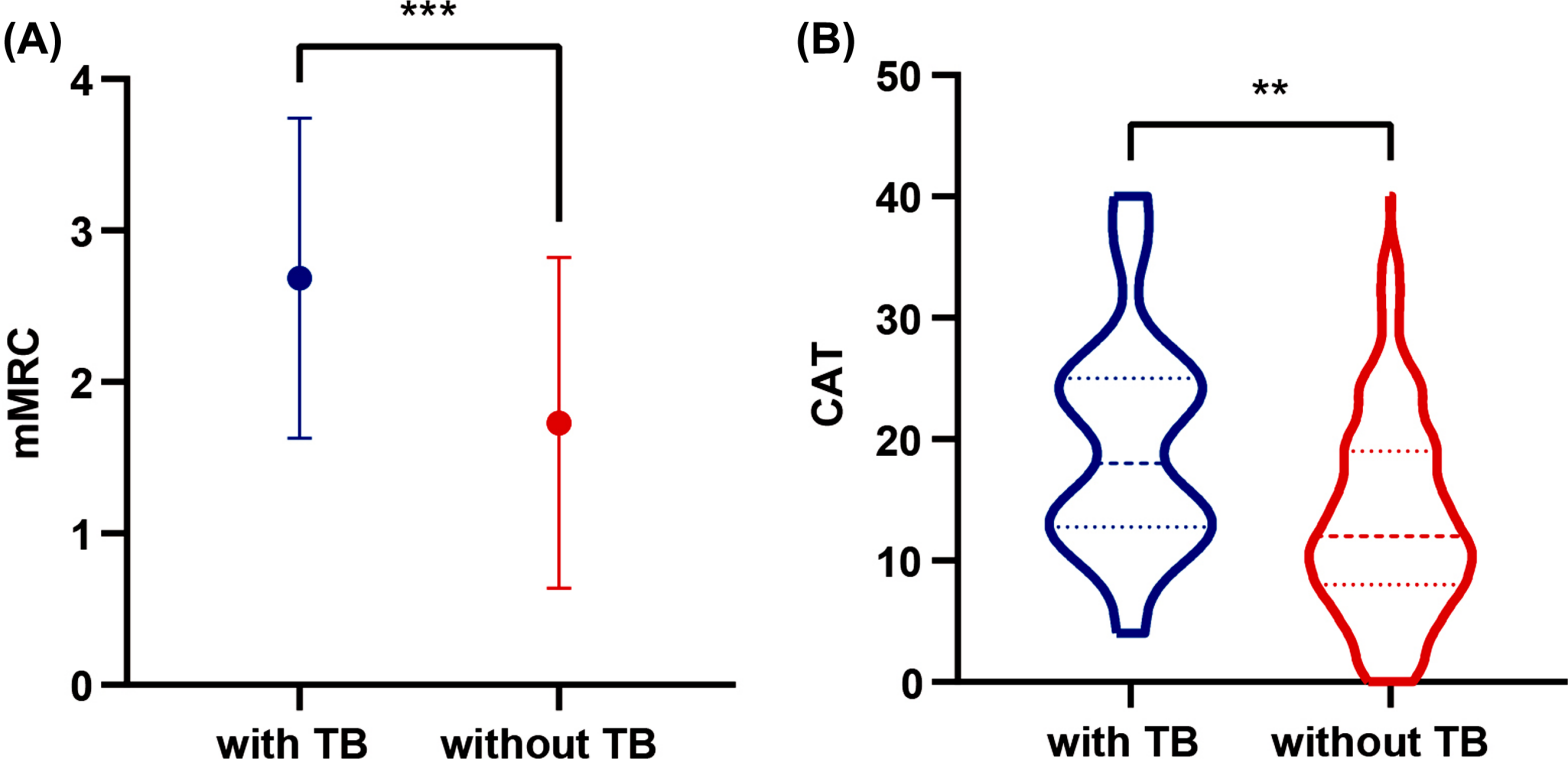
The association between a history of tuberculosis and symptom severity among COPD patients. **Notes:** Compared to the group with no history of tuberculosis, mMRC (**A**) and CAT (**B**) scores were higher in the history of tuberculosis group among COPD patients. **Abbreviations:** TB, pulmonary tuberculosis; mMRC, modified British medical research council; CAT, COPD assessment test.

In COPD patients, the mMRC in the prior TB group was higher than that in the non-TB group (Fig. **2A**), and the difference was statistically significant (*p*-value = 0.001). Compared to the control group of COPD patients, the CAT scores in the prior TB group were higher (Fig. **2B**), and the differences were statistically significant (*p*-value = 0.002).

### 3.4 The Associations between Prior TB and Exacerbations among COPD Patients

**Fig. (3):**
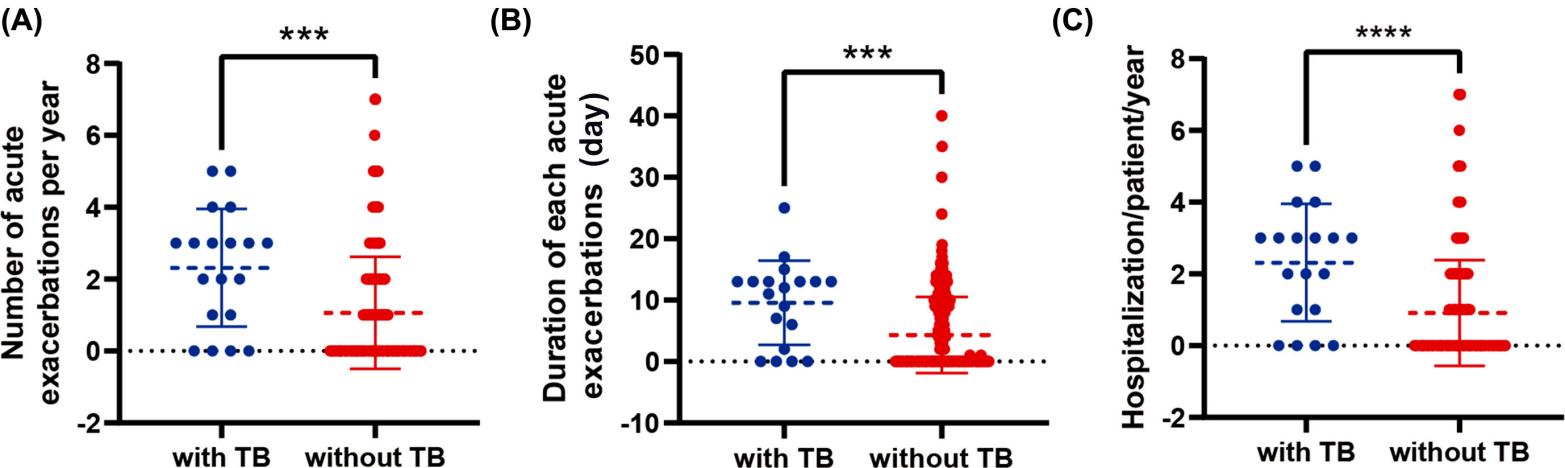
The association between a history of tuberculosis and acute exacerbations among COPD patients. **Notes:** Compared to the group with no history of tuberculosis, the number of acute exacerbations per year (**A**), the duration of acute exacerbations (**B**), and hospitalization/patient/year (**C**) were higher in the history of tuberculosis group among COPD patients. **Abbreviations:** TB, pulmonary tuberculosis.

The association between a history of tuberculosis and acute exacerbation has been observed. The index of the number of acute exacerbations per year in the prior TB group was higher than that in the non-TB group (Fig. **3A**), and the difference was statistically significant (*p*-values = 0.008). Compared to the control group involving COPD patients, the duration of acute exacerbation in the prior TB group was increased (Fig. **3B**), and the differences were statistically significant (*p*-values = 0.004). Moreover, hospitalization/ patient/year in the prior TB group was significantly higher than that in the non-TB group among COPD patients. (Fig. **3C**), and the difference was statistically significant (*p*-values<0.001).

### 3.5 The Relationship between the Probability of Developing COPD and FEV1/FVC (%) among Prior TB Patients

**Fig. (4):**
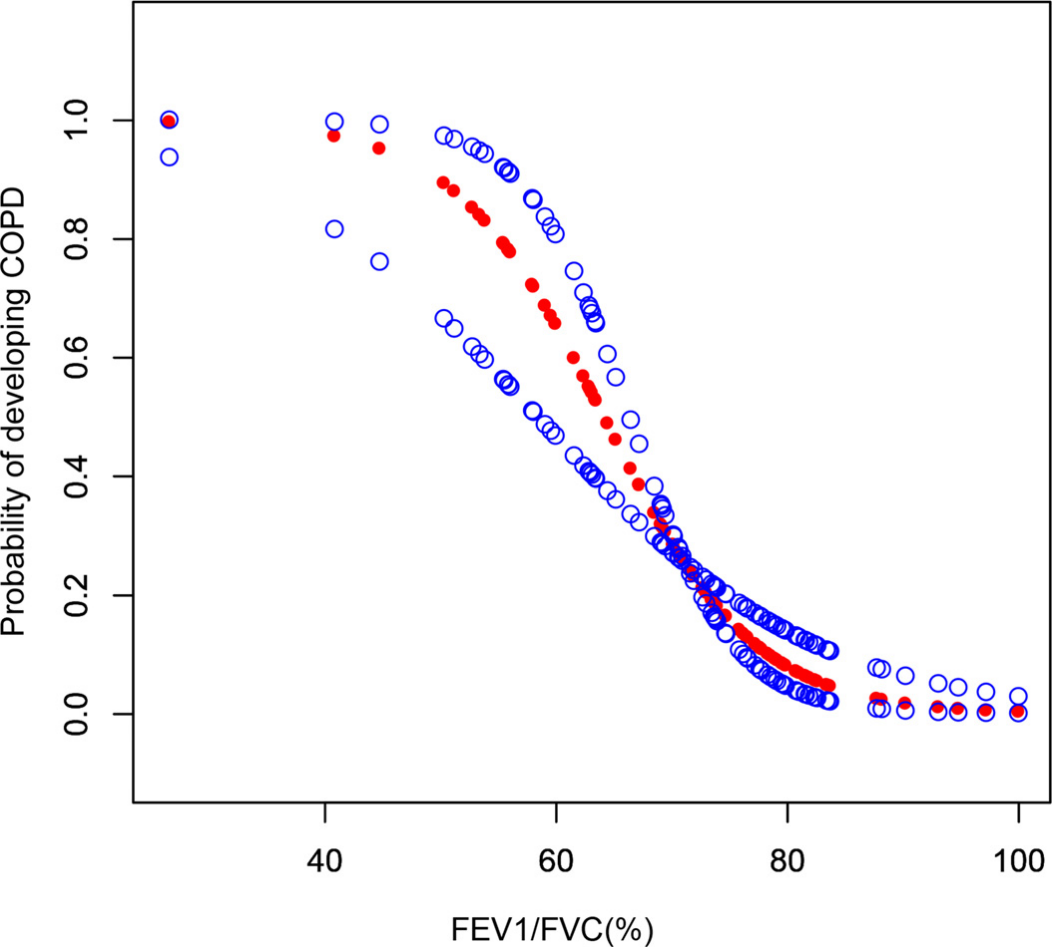
Dose-response relationship between the probability of developing COPD and lung function, FEV1/FVC (%), among pulmonary tuberculosis population. **Abbreviations:** FEV1, forced expiratory volume in 1 s; FVC, forced vital capacity; COPD, chronic obstructive pulmonary disease. **Notes:** Adjusted for age, economic income, education level, smoke exposure, and other vital covariates, including previous chronic bronchitis, diabetes, insomnia, *etc*.

There was a dose-response relationship between FEV1/FVC (%) and the probability of developing COPD in people with previous pulmonary TB (Fig. **4**). The piecewise regression analysis showed that the dose-response curve's cut-off point was FEV1/FVC (%) = 81.5. After adjusting for covariates, such as age, economic income, education level, smoke exposure, and other vital covariates, including previous chronic bronchitis, diabetes, and insomnia, a dose-response relationship was found between them and the cut-off point was FEV1/FVC (%) = 80.8. In the prior TB patients, when FEV1/FVC (%) was ≥ 80.8, the risk of COPD increased and was not significantly associated with lung function (*p*-value = 0.993). When FEV1/FVC (%) was < 80.8, the risk of COPD increased by 13.5% per unit decrease in lung function [0.865(0.805, 0.930)] (p-values < 0.001) (Table **2**).

## 4 DISCUSSION

PTB is a significant risk factor for COPD, and Xinjiang, China, belongs to the region with a high PTB burden [21-23]. The impact of the disease on COPD is not known locally. To our knowledge, this is the first study to examine the effects of a history of tuberculosis on airflow restriction, clinical symptoms, and acute episodes in patients with COPD in Xinjiang. Besides, studies on the relationship between lung function changes and the risk of COPD in the general population have been relatively conducted, and several clinical prediction models have shown that lung function is an essential predictor of COPD [11, 12]. However, the specific relationship between the two has not been reported in people with a TB history. In this study, we observed for the first time a dose-response relationship between pulmonary function changes and the probability of developing COPD in people with a history of TB, and the cut-off point was FEV1/FVC (%) = 80.8. In the prior TB patients, when FEV1/FVC (%) was ≥ 80.8, the risk of COPD increased but was not significantly associated with lung function. when FEV1/FVC (%) was < 80.8, the risk of COPD increased by 13.5% per unit decrease in lung function [0.865(0.805, 0.930)]. This study's results remind us that in countries with a high TB burden, it is necessary to enhance the dynamic detection of pulmonary function in people with a history of TB and implement early targeted interventions to prevent COPD's occurrence and progression.

Increasingly evidence shows that PTB is an important risk factor for COPD, particularly in nonsmoking populations [13, 24, 25]. In a vast number of developing countries, including China, the association between tuberculosis and COPD in terms of epidemiology requires us to more actively understand the relationship between the two in terms of morbidity, disease progression, and prognosis. The prevalence of tuberculosis in southern Xinjiang has been higher than in other parts of the country [21-23]. Our data showed that the prevalence of COPD among local TB patients was 21.8%, significantly higher than that in the control group (11.9%), and the difference was statistically significant. The risk of developing COPD in patients with TB was 2.181 times that of those without TB. These findings provide some epidemiological evidence to support tuberculosis as an essential risk factor for COPD among the local people.

A cross-sectional study involving 14,050 patients showed a significant positive association between tuberculosis history and airflow restriction after adjusting for confounders (adjusted odds ratio 2.13, 95% CI 1.42-3.19) [19]. Hnizdo, E *et al.* found that the frequency of acute tuberculosis attacks was related to airflow restriction frequency: the frequency of 1, 2, and more than 3 acute tuberculosis attacks corresponding to airflow restriction was 18.4%, 27.1%, and 35.2%, respectively [26]. Our results suggested that the prior TB group had more severe airflow limitation in both the general population and COPD patients than the non-TB group. The exact mechanism by which pulmonary tuberculosis causes airflow restriction remains unclear. Some studies have proposed that pulmonary tuberculosis will have scar formation in the process of lung tissue healing, which can lead to the loss of lung parenchymal tissue and cause the limitation of lung function [27]. It has also been suggested that lung macrophages may play an essential role in TB-induced airflow restriction. As important players in regulating host responses to injury and tissue repair, pulmonary macrophages can undergo intense inflammatory reactions and severe airway remodeling under the action of latent Mycobacterium tuberculosis [19, 28, 29]. TLR2 is a key molecule regulating the autophagy of alveolar macrophages [30]. After mycobacterium tuberculosis infection, the TLR2 signalling pathway can be activated to induce the secretion of CXCL5 by lung epithelial cells, which is an important link in the inflammatory response and airway remodelling driven by the polymorphonuclear leukocytes [31, 32]. In addition, Li, *et al*. found that genetic polymorphisms of RS10759932 and RS2737190 in TLR4 are significantly related to both COPD and PTB [33]. In addition, studies have found that the content of MMP-1, MMP-9, and other matrix metalloproteinases in bronchoalveolar lavage fluid of pulmonary tuberculosis patients is significantly increased [34, 35]. In particular, upregulation of MMP-1 expression can be involved in the progression of emphysema by regulating the MyD88/IRAK1 signalling pathway [36]. In addition, alveolar macrophages are an essential source of MMPs in emphysema. Therefore, it is of great significance to systematically study the specific roles of TB, alveolar macrophages, matrix metalloproteinases, and COPD to clarify the interaction mechanism between pulmonary tuberculosis and COPD.

Acute exacerbation of COPD is a clinical process characterized by an acute exacerbation of respiratory symptoms, such as dyspnea, cough, and increased sputum volume. At present, some scholars regard it as an independent disease phenotype of COPD [37, 38]. COPD's acute aggravation is very harmful, which accelerates the disease process and is an essential factor that seriously affects patients' quality of life and is also a necessary reason for increased COPD mortality [39, 40]. Many factors influence acute exacerbations of COPD, including previous acute exacerbations, health status, age, gender, and so on [41]. However, there are few reports on the relationship between TB history and COPD exacerbation. Park, *et al*. found that the prevalence of severe exactions among people with TB history was significantly higher than those in the control group (28.8% *vs*. 23.5%; 3.9% *vs*. 1.5%) [20]. Our study showed that the annual number of acute exacerbations, acute exacerbation duration, and the length of hospital stay/patient/year were significantly higher in the prior TB group than in the non-TB group. Therefore, it is an important task and challenge for us to systematically study the interaction mechanism between tuberculosis and acute exacerbation of COPD and then prevent acute exacerbation of COPD, even frequent exacerbations, to reduce its incidence and harm.

Low lung function predicts the risk of subsequent COPD. In our previous COPD risk prediction model in a typical rural area, the lung function index was included in the histogram as a significant predictor [12]. The relationship between lung function changes and COPD risk in the general population has been well studied. However, there is currently a lack of research on the relationship between lung function and the probability of developing COPD in people with a TB history. This study showed a dose-response relationship between lung function and COPD risk in people with a history of TB. After adjusting for confounders, this relationship remained. This dose-response curve shows that when FEV1/FVC1 (%) falls below a certain cut-off point, the risk of COPD increases rapidly. This reminds us of the need for dynamic lung function testing in people with a history of TB.

However, there are limitations to our study. First, the history of TB in the study was determined by a face-to-face questionnaire based on the self-reported previous pulmonary tuberculosis history. Data on specific types of tuberculosis, years of disease, and kind of medication were not available, and further stratified analyses were not performed. Second, our findings are based on baseline data from the early stage of a single-center cohort study, which is cross-sectional and has a limited sample size. Therefore, this study is only preliminary, and the specific conclusion needs to be further verified by large sample size and prospective study.

## CONCLUSION

In conclusion, Xinjiang, China, is a region with a high incidence of TB. This study complements the lack of relevant studies on the influence of TB history on airflow restriction, clinical symptoms, and acute exacerbations in COPD patients. Furthermore, for the first time, we observed a dose-response relationship between lung function and the probability of developing COPD in people with a history of TB, providing theoretical support to the importance of detecting dynamic lung function in high-risk groups, such as those with a history of TB, to prevent and reduce the occurrence and development of COPD in countries or regions with a high burden of tuberculosis.

## Figures and Tables

**Table 1:** Baseline characteristic of study population.

**Demographic Characteristics**	**Total** **(*n*=3249)**	**PTB**		***P*-value**
		**Yes(*n*=87)**	**No(*n*=3162)**	
Age, year	52.8±9.4	56.9±9.9	52.7±9.3	<0.001
Sex	-	-	-	-
Male	1585(48.8%)	43(49.4%)	1542(48.8%)	0.903
Female	1664(51.2%)	44(50.6%)	1620(51.2%)	
BMI(kg/m^2^)	25.5±4.2	25.1±4.7	25.8±8.9	0.480
Waist, cm	92.9±11.4	90.4±15.7	93.0±11.7	0.044
Education Level	-	-	-	-
Illiterate	432(13.3%)	21(24.1%)	411(13.0%)	0.003
Primary or Higher	2817(86.7%)	66(75.9%)	2751(87.0%)	
Marital Status	-	-	-	-
Married	2781(85.6%)	79(90.8%)	2702(85.5%)	0.161
Divorce and so on	468(14.4%)	8(9.2%)	460(14.5%)	
Gross Annual Income, Yuan	-	-	-	-
<10000	1783(45.1%)	58(66.7%)	1725(54.6%)	0.025
≥10000	1466(54.9%)	29(33.3%)	1437(45.4%)	
Alcohol Intake Status	-	-	-	-
Yes	222(6.8%)	3(3.4%)	219(6.9%)	0.205
No	3027(93.2%)	84(96.6%)	2943(93.1%)	
Barbecue	-	-	-	-
Yes	472(14.5%)	12(13.8%)	460(14.5%)	0.844
No	2777(85.5%)	75(86.2%)	2702(85.5%)	
Smoking	-	-	-	-
Yes	462(14.2%)	7(8.0%)	455(14.4%)	0.095
No	2787(85.8%)	80(92.0%)	2707(85.6%)	
Passive Smoking	-	-	-	-
Yes	258(7.9%)	3 (3.4%)	255(8.1%)	0.116
No	2991(92.1%)	84 (96.6%)	2907(91.9%)	
Main Source of Energy	-	-	-	-
Biomass or Coal	3104(95.5%)	80 (92.0%)	3024 (95.6%)	0.101
Electricity or Gas	145(4.5%)	7 (8.0%)	138 (4.4%)	
Ventilation System	-	-	-	-
Yes	561(17.3%)	14 (16.1%)	547 (17.3%)	0.769
No	2688(82.7%)	73 (83.9%)	2615 (82.7%)	
Stove	-	-	-	-
Yes	2985(91.9%)	79 (90.8%)	2906 (91.9%)	0.711
No	264(8.1%)	8 (9.2%)	256 (8.1%)	
Physical Exercise	-	-	-	-
Yes	187(5.8%)	5 (5.7%)	182 (5.8%)	0.997
No	3062(94.2%)	82 (94.3%)	2980 (94.2%)	
Insomnia	-	-	-	-
Yes	2048 (63.0%)	54 (62.1%)	1994 (63.1%)	0.850
No	1201 (36.9%)	33 (37.9%)	1168 (36.9%)	
Diabetes	-	-	-	-
Yes	132 (4.1%)	7 (8.0%)	125 (4.0%)	0.056
No	3117 (95.9%)	80 (92.0%)	3037 (96.0%)	
Cor Pulmonale	-	-	-	-
Yes	42 (1.3%)	3 (3.4%)	39 (1.2%)	0.071
No	3207 (98.7%)	84 (96.6%)	3123 (98.8%)	
Chronic Bronchitis	-	-	-	-
Yes	521 (16.0%)	23 (26.4%)	498 (15.7%)	0.007
No	2728 (84.0%)	64 (73.6%)	2664 (84.3%)	
Depression	-	-	-	-
Yes	26 (0.8%)	3 (3.4%)	23 (0.7%)	0.005
No	3223 (99.2%)	84 (96.6%)	3139 (99.3%)	
COPD	-	-	-	-
Yes	394 (12.1%)	19 (21.8%)	375 (11.9%)	0.005
No	2855 (87.9%)	68 (78.2%)	2787 (88.1%)	

**Abbreviations:** PTB, pulmonary tuberculosis; BMI, body mass index; COPD, chronic obstructive pulmonary disease.

**Notes:** Data are presented as mean ± SD or number (%). *P*-values <0.05, verses control. *P*-values are calculated by Student’s t-test or chi-squared test as appropriate.

**Table 2:** The relationship between Probability of developing COPD and Lung function FEV1/FVC (%) in pulmonary tuberculosis population.

**-**		**COPD(Unadjusted)**	**-**	**-**	**COPD(Adjusted)**	**-**
**FEV1/FVC (%)**		**OR (95%CI)**	***P*-value**	**-**	**OR (95%CI)**	***P*-value**
-	<81.5	0.852(0.784, 0.925)	<0.001	<80.8	0.865(0.805, 0.930)	<0.001
-	≥81.5	0.000(0.000, Inf)	0.995	≥80.8	0.000(0.000, Inf)	0.993

**Abbreviations:** FEV1, forced expiratory volume in 1 s; FVC, forced vital capacity; COPD, chronic obstructive pulmonary disease.

**Notes:** Adjusted for age, economic income, education level, smoke exposure and other vital covariates including previous chronic bronchitis, Diabetes, Insomnia, *etc*.

## Data Availability

The data that support the findings of this study are available from the corresponding author [FL], upon reasonable request.
